# Is This a Blunder?

**Published:** 1895-12

**Authors:** 


### IS THIS A BLUNDER?

WE are reliably informed by a Kings county veterinarian that the bill passed at the recent session of the New York Legislature exempting veterinarians from jury duty does not apply to to Kings and Queens counties. By whose blunder does this state of affairs exist? Are the veterinarians of these two great counties less busy than those of sister counties of that Commonwealth? Are the live-stock interests of less value and importance in these large centres? Rise up, O'Shea, and explain!

One of the most grateful signs of the times comes to the veterinary profession in the rapid appreciation of the people throughout the country in according prominent places to many members of our calling at meetings, conventions, and gatherings of our most enlightened people, called together for the consideration of sanitary questions and those involving the animal-food supply.

---

Secretary Morton, in his annual report, makes a very strong point on the question of bearing the cost of veterinary inspection of animal products destined for foreign shipment. He well says that this surveillance adds an additional value to these products, enhances the returns to the shippers, and therefore should be paid for by them. This plan of government inspection was originally created in the interests of the agriculturist and stockraiser, to enhance the value of the products of the farm, but since almost the entire foreign meat-industry has become concentrated and controlled by a half-dozen or more men in the whole country it has become a mere bonus to these shippers, and, like many other concessions given under the guise of protection to infantile industries, has encouraged the growth of combines and monopolies that were never intended to be fostered by our parental government. He suggests that this work should become the province of the State or municipal government or boards of health, and the cost placed upon those who derive therefrom the immediate benefit.

---

The agitation of Dr. J. H. Wattles for a pure milk-supply in Kansas City, Mo., resulting in the creation of a milk-inspection that does not inspect, promises to be a farce, unless a better state of affairs is created. When will citizens learn that questions involving the health and comfort of their people must be in every way separated from a political conception in their solution? Of what use is milk-inspection by a layman, and that only from a commercial point of view, with penalties that dwarf the fear of punishment into insignificance; inspection that leaves the sources of production unquestioned and utterly fails to create a single barrier of protection from the serious dangers that surround its production?

